# Iodoacetamine-Alkyne Derivatization-Based Liquid Chromatography–Mass Spectrometry Method for Quantification of Thiol Metabolites in Serum Samples of Hepatocellular Carcinoma Patients

**DOI:** 10.3390/metabo16050345

**Published:** 2026-05-20

**Authors:** Chun Mei, Xin-Ze Wu, Hua-Ming Xiao, Azamat Temerdashev, Na An, Quan-Fei Zhu, Yu-Qi Feng

**Affiliations:** 1 Department of Chemistry, Wuhan University, Wuhan 430072, China; chunmei@whu.edu.cn (C.M.); wuxinze@whu.edu.cn (X.-Z.W.); 2 School of Biomedical Engineering and Health, Wuhan Textile University, Wuhan 430200, China; naan_anna@whu.edu.cn (N.A.); qf_zhu@whu.edu.cn (Q.-F.Z.); 3 Analytical Chemistry Department, Kuban State University, Krasnodar 350040, Russia; TemerdashevAZ@gmail.com

**Keywords:** hepatocellular carcinoma, thiol metabolite, chemical derivatization, iodoacetamine-alkyne, liquid chromatography-mass spectrometry

## Abstract

**Background/Objectives**: The dysregulation of thiol metabolites is strongly linked to hepatocellular carcinoma (HCC) pathogenesis. However, quantifying these highly polar and oxidation-prone thiols in clinical serum samples via conventional liquid chromatography–mass spectrometry (LC-MS) remains challenging due to their poor sensitivity and reproducibility. **Methods**: We developed a sensitive and robust iodoacetamine-alkyne (IAM) derivatization–based LC-MS method for quantification of seven trans-sulfuration pathway thiols in human serum. **Results**: IAM derivatization markedly improved the method’s specificity due to enhanced chromatographic retention and diagnostic MS/MS fragments containing both the alkyne tag and analyte backbone. Sensitivity increased 33-to-160-fold versus underivatized analytes, with limits of detection of 0.02–0.1 nM. All analytes exhibited good linearity, acceptable precision with intra-day and inter-day relative standard deviations in the range of 1.2–13.8%, and high recovery from 88.6% to 102.9%. **Conclusions**: From the thiol quantification in human serum from 40 HCC patients and 40 healthy controls, it was found that levels of cysteine, homocysteine, glutathione, and cysteinylglycine were significantly lower in HCC patients (*p* < 0.05). A two-variable logistic regression model using cysteine and cysteinylglycine achieved 90.0% specificity and 80.0% sensitivity for robust HCC discrimination between HCC patients and healthy controls to some extent, with an area under the receiver operating characteristic curve of 0.88 (95% confidence interval: 0.792–0.968).

## 1. Introduction

Hepatocellular carcinoma (HCC) is responsible for the third largest burden of cancer-related mortality worldwide [1,2]. Early diagnosis remains challenging due to its silent onset, lack of distinctive symptoms, and limited availability of highly specific biomarkers [1,2,3,4]. Recent studies have revealed that HCC development and progression are accompanied by profound metabolic reprogramming, with widespread dysregulation of redox homeostasis and sulfur metabolism [3,4,5]. The trans-sulfuration pathway links the methionine cycle to de novo cysteine synthesis [5,6]. It helps maintain balance among seven thiol-containing metabolites including homocysteine (Hcy), cysteine (Cys), glutathione (GSH), γ-glutamylcysteine (Glu-Cys), cysteinylglycine (Cys-Gly), N-acetylcysteine (NAC), and cysteamine (CA) [6,7]. Within this pathway, Hcy is converted to Cys via vitamin B_6_-dependent cystathionine β-synthase (CBS) [5,6,7]. Cys functions both as the rate-limiting substance in GSH biosynthesis and as a metabolic precursor that generates bioactive metabolites such as NAC and Cys-Gly through deacetylation or hydrolysis [6,7]. CA, in turn, modulates redox signaling through the regulation of protein thiol modifications. They form a reactive sulfur metabolic network that reflects hepatic oxidative stress and metabolic adaptability, making them promising candidate biomarkers.

However, all seven metabolites are naturally present in human serum at micromolar levels [8,9,10]. Analysis of these seven thiol-containing metabolites (RSHs) within the trans-sulfuration pathway by liquid chromatography–mass spectrometry (LC-MS) remains challenging [11,12,13,14]. Free thiols are highly susceptible to oxidation under physiological pH and ambient temperature, resulting in underestimation and poor analytical reproducibility [9,10,11,15]. Moreover, RSH compounds exhibit high polarity and low hydrophobicity, leading to poor retention on reversed-phase liquid chromatography and diminished electrospray ionization efficiency [10,12,13,15,16,17,18]. Several RSH species possess structural isomers and contain multiple reactive functional groups. Chemical derivatization represents a widely adopted strategy to overcome these limitations [11]. However, few reagents currently enable efficient and selective derivatization of all seven RSH analytes. Existing reagents, including N-ethylmaleimide (NEM) [19], iodoacetamide (IAA) [20], and ω-bromoacetylquinoline bromide (BQB) [8,10], are associated with drawbacks such as extensive side reactions, insufficient site selectivity, incomplete derivatization, severe interference from polar matrix components (necessitating additional complex sample pretreatment steps), and suboptimal mass spectrometric specificity [18]. Thus, the comprehensive quantification of thiol metabolites in the trans-sulfuration pathway still needs further evaluation, especially in human serum samples.

**Figure 1:**
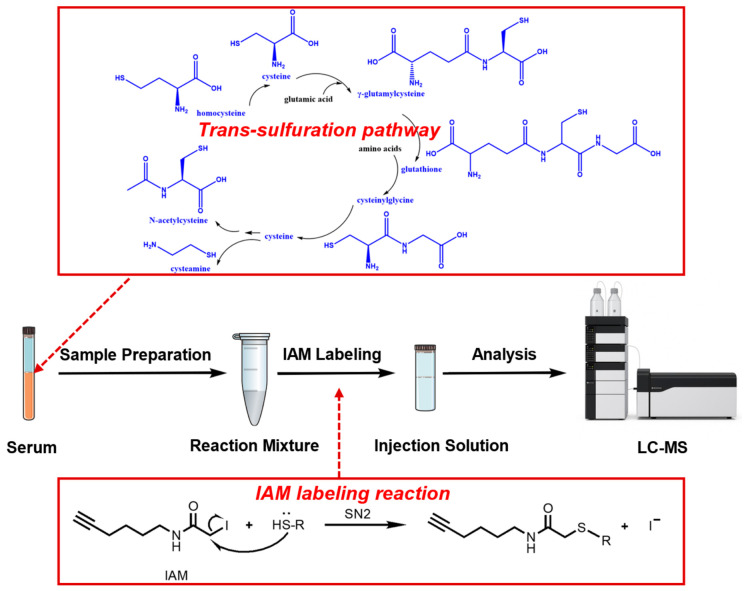
Schematic diagram of quantitation of thiols by IAM-LC-MS method, inserted trans-sulfuration pathway (thiols marked blue) and reaction schematic of IAM towards thiols.

Given the extremely low endogenous abundance of alkyne-containing metabolites in humans and the steric inertness of the alkyne group [21,22,23,24,25], we speculated that selective detection of alkyne-tagged analytes in the multiple reaction monitoring (MRM) mode of LC-MS/MS would achieve two key advantages, including minimizing nonspecific consumption of the derivatization reagent during sample processing and effectively filtering out high-abundance matrix interferences (such as abundant lipids), thereby substantially improving the method’s selectivity in human serum samples. Accordingly, we selected iodoacetamine-alkyne (IAM) as a thiol-selective derivatization reagent (Figure 1). We systematically evaluated IAM for its labeling specificity, enhancement of chromatographic retention, consistency of MS/MS fragmentation patterns, and detection sensitivity. We also ran parallel experiments for direct comparison with BQB, previously developed in our group [8,10]. Leveraging the alkyne tag of IAM, we successfully established a robust, enrichment-free, operationally simple LC-MS/MS method for thiol-containing metabolite quantification in the trans-sulfuration pathway, featuring both high sensitivity and high specificity (Figure 1). This method was subsequently employed to compare serum samples from individuals diagnosed with HCC and healthy controls (CTLs).

## 2. Materials and Methods

### 2.1. Chemical Reagents

Thiol standards including CA, Cys, Hcy, NAC, and reduced GSH were purchased from Sigma-Aldrich (St. Louis, MO, USA). Cys-Gly, deuterated HCy (d4-Hcy), and Glu-Cys were obtained from Shanghai Yuanye Bio-Technology Co., Ltd. (Shanghai, China). Amino standards (n-decylamine (DA), cyclohexylamine (CHA), and 2,6-dimethylaniline (2,6-DTA)) and carboxylic acid standards (n-hexanoic acid (HexA), ricinoleic acid (RA), and benzoic acid (BA)) were purchased from Aladdin Biochemical Technology Co., Ltd. (Shanghai, China). IAM was purchased from Bide Pharmatech Ltd. (Shanghai, China). Formic acid (FA, ≥98% purity), ethylenediaminetetraacetic acid disodium salt (EDTA-2Na, ≥99% purity), and tris(2-carboxyethyl)phosphine (TCEP) were obtained from Sinopharm Chemical Reagent Co., Ltd. (Shanghai, China). Ultrapure water was prepared using a Milli-Q water purification system (Millipore, MA, USA). Acetonitrile (LC-MS grade) was supplied by FTSCI Corp. (Wuhan, China). All thiol-containing metabolites were dissolved in 1 mmol/L EDTA-2Na solution containing 0.05% (*v*/*v*) formic acid to prepare 10 mmol/L stock solutions and then stored at −20 °C in the dark until use.

### 2.2. Collection of Serum Samples

In total, 40 serum specimens from healthy individuals and another 40 from patients with a clinical diagnosis of HCC were collected from Zhongnan Hospital, Wuhan University (Wuhan, China). Written informed consent was obtained from all participants before blood collection. The study design and biospecimen management protocols adhered rigorously to the ethical standards stipulated in the Declaration of Helsinki. Ethical approval for this study was granted by the Medical Ethics Committee of Zhongnan Hospital of Wuhan University (Ethics Approval No. 2017058). All serum samples were stored at −80 °C to ensure stability.

### 2.3. Extraction, Derivatization, and LC-MS Analysis of Thiol Metabolites in Human Serum Samples

Twenty microliters of serum were transferred into a 1.5 mL centrifuge tube. Then, 75 µL of 1.0 mmol/L EDTA-2Na solution containing 0.5% (*v*/*v*) formic acid and 5 µL of 10 mmol/L TCEP solution were added. The mixture was vortexed and incubated at 45 °C for 1 h at 1500 rpm to reduce the disulfide bonds and chelate the metal ions. Then, 200 µL of ACN was added and the resulting mixture was kept at 4 °C for 1 h to precipitate the proteins. It was then centrifuged at 12,000 rpm for 5 min at 4 °C. The supernatant was collected and dried under a stream of nitrogen gas. The residue was reconstituted in 100 µL of 1.0 mmol/L EDTA-2Na solution containing 0.5% (*v*/*v*) formic acid. This solution was used for subsequent analysis.

For IAM derivatization, 20 µL of 100 mM IAM solution, 50 µL of 2 mM phosphate buffer (pH 6.5), 10 µL of 2 µM d4-Hcy (internal standard), and 20 µL of the above serum extract were added sequentially to a 1.5 mL centrifuge tube. The mixture was vortexed and incubated at 50 °C for 4 h at 1500 rpm in the dark. After incubation, the reaction mixture was injected directly into the LC-MS system. BQB derivatization was performed following the method in a previous publication [8].

LC-MS analysis was performed on a Shimadzu LC-MS 8050 system equipped with an electrospray ionization source and operated in MRM mode. Chromatographic separation was achieved using a Waters Acquity UPLC BEH C18 column (50 mm × 2.1 mm, 1.7 µm). The mobile phase consisted of 0.1% formic acid in water (A) and acetonitrile (B). A gradient elution program was applied as follows: 0–1 min, 5% B; 1–5 min, 5–50% B; 5–6 min, 50–90% B; 6–7 min, 90% B; 7–8 min, 90–5% B; 8–12 min, 5% B. The flow rate was 0.4 mL/min. The column temperature was maintained at 40 °C and the injection volume was 2 µL. All derivatized analytes were quantified in positive-ion MRM mode. The characteristic precursor-to-product ion transitions and corresponding collision energies are listed in Table S1. The ion source parameters were set as follows: desolvation temperature, 400 °C; heat block temperature, 250 °C; interface temperature, 300 °C; nebulizing gas flow, 3 L/min; drying gas flow, 10 L/min; and heating gas flow, 10 L/min. Data acquisition and processing were performed using LabSolutions software (v5.5 SP2).

### 2.4. Method Performance Evaluation

A calibration curve comprising 15 concentration levels was constructed across the specified analytical range of 0.1–5000 nmol·L^−1^ (0.1, 0.2, 0.5, 1, 2, 5, 10, 20, 50, 100, 200, 500, 1000, 2000, and 5000 nmol·L^−1^). Each calibration standard contained 200 nmol·L^−1^ d4-Hcy as an internal standard (IS). After derivatization under optimized conditions, samples were subjected to LC-MS/MS analysis. A linear regression equation was generated by plotting the peak area ratio (analyte/IS) against the nominal concentration. The limit of detection (LOD) and limit of quantification (LOQ) were defined as the lowest concentrations yielding signal-to-noise (S/N) ratios of 3 and 10, respectively. Recovery and matrix effects were evaluated in a human serum matrix for Cys, Hcy, GSH, NAC, Cys-Gly, Glu-Cys, CA, and the internal standard d4-Hcy. Three parallel sample sets were prepared: (A) matrix spiked with IAM derivatives, (B) analytes spiked before extraction, (C) analytes spiked after extraction, and (D) unspiked matrix. The extraction recovery (ER, %) was calculated as the peak area ratio from (B/C) × 100. The matrix effect (ME, %) was calculated as the peak area ratio from (A/D) × 100. The method’s precision and accuracy were assessed using human serum samples spiked at three concentration levels (1, 50, and 100 nmol·L^−1^). Intra-day precision was determined by analyzing five replicates of each concentration on a single day. Inter-day precision was determined by analyzing three replicates per concentration on each of three consecutive days (total *n* = 9). Accuracy was expressed as the percentage recovery, calculated as (measured concentration/nominal spiked concentration) × 100.

### 2.5. Statistical Analysis

Data normality was assessed using the Shapiro–Wilk test. Group differences between the HCC and CTL groups were evaluated using the Mann–Whitney U test. Variables with significant differences were further subjected to LASSO regression for feature selection, followed by univariate and multivariate logistic regression analyses to construct diagnostic models. Partial Least Squares Discriminant Analysis (PLS-DA) multivariate analyses were used to explore metabolic profile separation between groups. Model fit and robustness were assessed via cross-validation and permutation testing. Score plots and permutation test plots were generated to evaluate group discrimination and model reliability. Receiver operating characteristic (ROC) curves were constructed and the area under the curve (AUC) was calculated to assess the diagnostic performance of individual biomarkers and combined models. Boxplots, linear trend plots, extracted ion chromatograms (EICs) from MRM data, and optimization-related figures were generated using professional statistical and visualization software. All statistical tests were two-tailed, and *p* < 0.05 was considered statistically significant. Significance levels are indicated in the figures as follows: * *p* < 0.05, ** *p* < 0.01, and *** *p* < 0.001.

## 3. Results

### 3.1. Chemical Reaction Between IAM and RSH

The IAM undergoes an SN2 nucleophilic substitution reaction with thiol groups to yield stable thioether derivatives (Figure 1). In principle, IAM may also react with amino or carboxyl groups. To systematically evaluate its reaction selectivity, we selected three thiol-containing compounds (Hcy, NAC, and GSH), three amino-containing compounds (DA, CHA, and 2,6-DTA), and three carboxylic acid compounds (HexA, RA, and BA) as model compounds. Under phosphate-buffered conditions, IAM exhibited pronounced pH-dependent reactivity toward thiol compounds (Figure S1). At pH 2.5–3.5, derivative signals for Hcy, NAC, and GSH were weak; signal intensities increased sharply with rising pH and plateaued at pH 6.5. Notably, at pH > 8.0, a distinct derivative signal emerged for 2,6-DTA, indicating that amino groups can react with IAM under alkaline conditions. In contrast, no derivative signals were detected for any of the carboxylic acid compounds across the entire pH range tested (2.5–10.0), confirming that IAM does not react with carboxyl groups under these conditions. Although Hcy, NAC, and GSH contain both thiol and either amino or carboxyl functionalities, no multi-site derivatization products arising from concurrent reaction at multiple functional groups were observed under near-neutral conditions (pH 6.5–7.5). Collectively, the results demonstrate that IAM enables selective thiol labeling under near-neutral conditions. Considering that thiols retain sufficient nucleophilicity while remaining relatively resistant to oxidation-induced disulfide formation at a mildly acidic to neutral pH, we selected pH 6.5 as the optimal condition for subsequent derivatization optimization.

We further investigated the effects of reaction temperature (40–80 °C), reaction time (0.5–10 h), and IAM concentration (1–40 mM) on derivatization efficiency (Figure S1). Results indicated that derivatization yield reached a plateau under the following conditions: 50 °C for 4 h with 10 mM IAM. Moreover, the derivatized products exhibited excellent stability: when stored at room temperature for 24 h, their peak areas showed a relative standard deviation (RSD) < 10%.

### 3.2. Comparative Analysis of IAM and BQB Reagents Towards RSH

**Figure 2:**
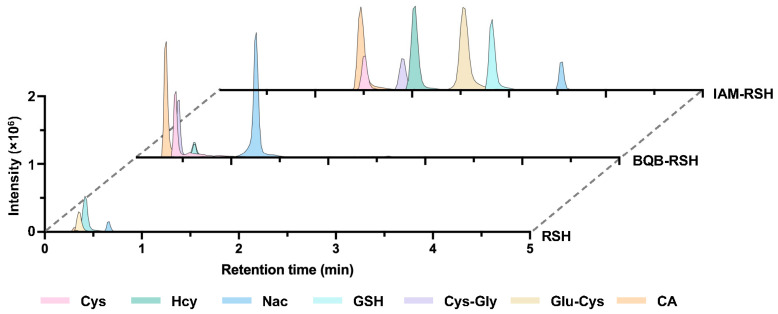
Chromatograms of underivatized RSH, BQB derivatives of RSH (BQB-RSH), and IAM derivatives of RSH (IAM-RSH).

We conducted a comprehensive comparison between IAM and BQB across multiple analytical dimensions, including chromatographic retention behavior, MS/MS fragmentation patterns, and detection sensitivity. Under identical chromatographic conditions, underivatized thiol compounds exhibited weak retention and were highly susceptible to co-eluting matrix interference. Upon labeling with the permanently positively charged BQB reagent, most thiol analytes (except NAC) showed markedly reduced retention times and broadened peak shapes (Figure 2). In contrast, IAM-derived products displayed stronger retention (Figure 2), thereby facilitating partial separation from highly polar endogenous metabolites.

**Figure 3:**
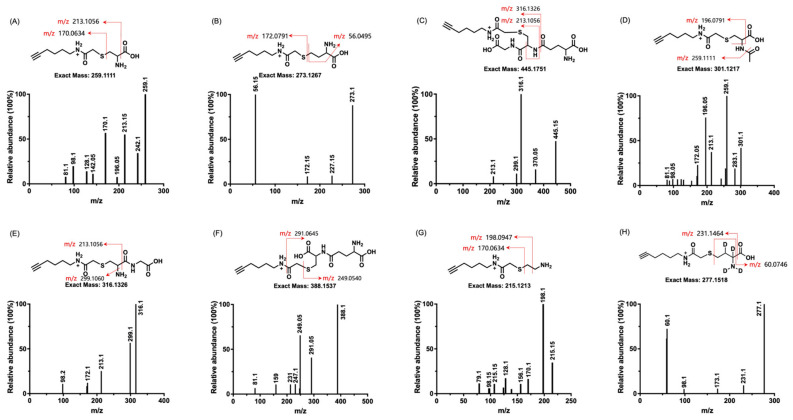
MS/MS spectra of IAM derivatives including (**A**) Cys, (**B**) Hcy, (**C**) GSH, (**D**) NAC, (**E**) Cys-Gly, (**F**) Glu-Cys, (**G**) CA, and (**H**) d4-Hcy with 20 V collision energy.

In MS/MS analysis, BQB-labeled derivatives consistently generated two characteristic fragment ions (*m*/*z* 130 and *m*/*z* 218) derived from the reagent moiety [8,10]. By contrast, IAM-labeled derivatives (Figure 3) predominantly underwent cleavage at the thioether bond (C–S), yielding fragments containing the analyte backbone, and/or at the C–N bond adjacent to the amino terminus of the thiol moiety. Additionally, decarboxylation (neutral loss of CO_2_) was also observed as a diagnostic feature. For example, cysteine (Cys) and homocysteine (Hcy) yielded fragment ions at *m*/*z* 170 (C–S cleavage) and *m*/*z* 213 (CO_2_ loss) and at *m*/*z* 56/172 (C–S cleavage) and *m*/*z* 227 (CO_2_ loss), respectively. Unlike the quinoline-ring-derived fragments (*m*/*z* 130) observed for BQB, IAM-generated fragments retained the alkyne moiety covalently linked to the thiol moiety. In MRM-based quantification, although BQB labeling substantially enhances the ionization efficiency of thiol compounds, the selected MRM transitions for the BQB derivatives originate exclusively from reagent-specific fragment ions. In contrast, for the IAM derivatives, the MRM transitions are defined by fragment ions comprising both the alkyne tag and the thiol moiety (Figure 3, Table S1). Compared with underivatized thiols, IAM derivatization thereby improved detection sensitivity by 33-to-160-fold, achieving limits of detection (LODs) of 0.02–0.1 nM (Table S2). Relative to BQB, IAM provided 2-to-16.7-fold higher MRM sensitivity for all tested compounds except NAC. These findings underscore that the incorporation of the alkyne tag into the analyte-derived fragment ion is the key determinant underlying the superior MRM sensitivity of IAM-based derivatization.

### 3.3. Method Validation

To systematically evaluate the analytical performance of the IAM derivatization strategy in the serum matrix, we assessed the linearity, accuracy, extraction recovery, and matrix effects. For multiple thiol metabolites exhibiting substantial structural diversity, the use of analyte-specific stable isotope-labeled internal standards provides superior quantitative correction. However, considering the limited commercial availability of such isotopically labeled standards and the need to reduce operational costs in clinical analysis, we explored the feasibility of using d4-Hcy as the single IS to correct for systematic bias across the entire analytical workflow and subsequently conducted comprehensive method validation. Results confirmed that there is no carry-over effect observed in the method. Good linearity for all seven thiol compounds over the range of 0.1–5000 nmol·L^−1^ (R^2^ ≥ 0.9981) was found when d4-Hcy was employed as the IS. The limits of detection (LODs) ranged from 0.02 to 0.1 nM (Table 1), confirming high sensitivity. The intra-day RSDs were 0.7–8.9% and the inter-day RSDs were 1.5–10.7% at three levels of concentration. The extraction recoveries and matrix effects were found to be 86.2–113.6% and 82.4–108.9%, respectively (Table 2, Figure S2). These results demonstrate that the method exhibits a wide linear range, high sensitivity, good precision and accuracy, and robust tolerance to the serum matrix, making it suitable for reliable thiol metabolite quantification. In addition, the established method was compared with existing assays for RSH determination with respect to both the number of thiols detected and their LODs (Table 3). Compared to published methods for detecting the thiols in the trans-sulfuration pathway, our method achieves the lowest LODs for LC-MS analysis without requiring complex enrichment steps.

### 3.4. Thiol Levels in Human Serum of HCC Patients and Health Controls

To assess the practical utility of the IAM derivatization strategy in complex biological samples, we applied the established LC-MS targeted metabolomics method to quantify the seven thiol metabolites in serum samples from 40 HCC patients and 40 healthy controls (CTLs) (Table S3). All samples were stratified by age and sex and randomly assigned in a 3:1 ratio to a discovery set (n = 60, HCC/CTL = 30/30) and an independent validation set (n = 20, HCC/CTL = 10/10) to minimize confounding bias and ensure statistical robustness in model development and validation.

**Figure 4:**
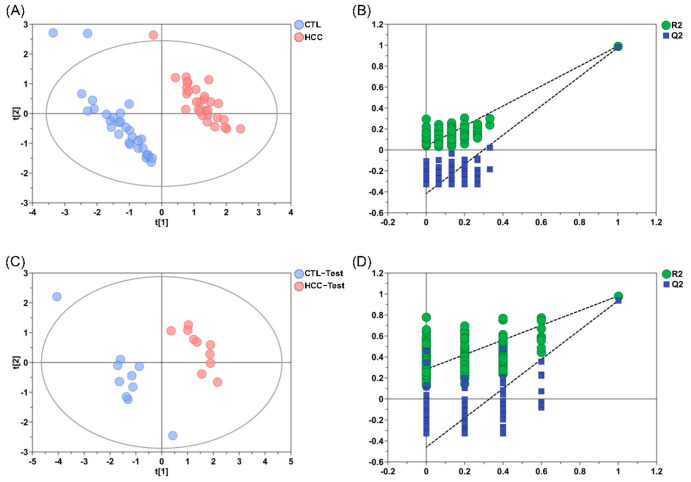
PLS-DA analysis of seven thiol metabolites in HCC patients versus healthy controls including (**A**) PLS-DA score plot for the discovery set, (**B**) permutation test plot (200 permutations) of the PLS-DA model for the discovery set, (**C**) PLS-DA score plot for the validation set, (**D**) permutation test plot (200 permutations) of the PLS-DA model for the validation set.

**Figure 5:**
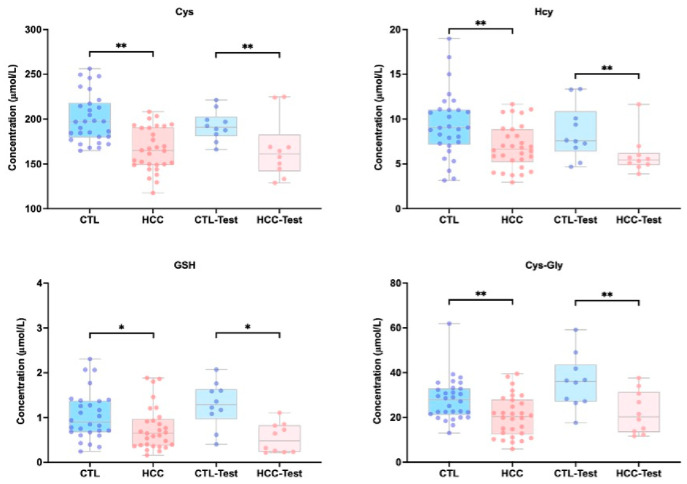
Concentration distributions of Cys, Hcy, GSH, and Cys-Gly in serum samples from HCC and healthy controls (CTLs) within both the discovery and validation cohorts. Statistical significance was assessed using the Mann–Whitney U test: * *p* < 0.05, ** *p* < 0.01.

**Figure 6:**
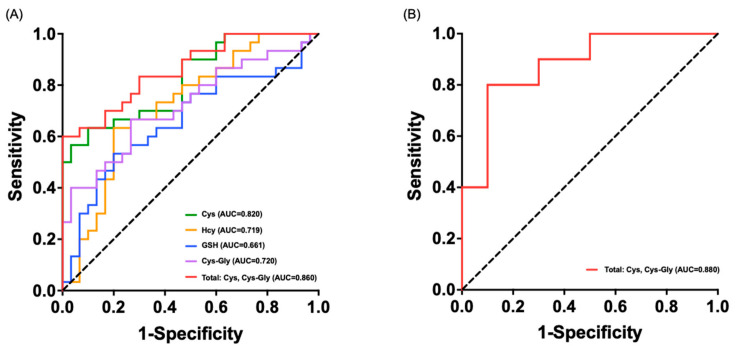
(**A**) ROC curves of Cys, Hcy, GSH, Cys-Gly, and the combined Cys and Cys-Gly model in the discovery set, (**B**) ROC curve of the combined Cysand Cys-Gly diagnostic model in the validation set.

A PLS-DA model built from the quantitative data showed clear and highly consistent separation between the HCC and CTL groups in both the discovery and validation sets (Figure 4). A 200-permutation test indicated strong model fit, reliable predictive power, and resistance to overfitting. Mann–Whitney U tests revealed significantly lower levels of Cys, Hcy, GSH, and Cys-Gly in the HCC group versus CTLs (*p* < 0.05), and this difference was fully replicated in the validation set (Figure 5, Tables S4 and S5). The largest decreases were observed for Cys (*p* < 0.01) and GSH (*p* < 0.01), reflecting depletion of intracellular reduced glutathione and increased oxidative stress. Concurrent reductions in Hcy and Cys-Gly suggest impaired activity of key trans-sulfuration enzymes (CBS, CTH) and insufficient supply of glutathione precursors. Together, these coordinated changes form a characteristic metabolic pattern, indicating systemic suppression of thiol metabolism in HCC, with clear pathophysiological relevance and clinical discrimination potential. ROC analysis showed Cys had the highest single-metabolite discriminative power (AUC = 0.820) (Figure 6A). Cys-Gly (AUC = 0.720), Hcy (AUC = 0.719), and GSH (AUC = 0.661) also achieved statistical significance (*p* < 0.01) (Figure 6A). A two-metabolite logistic model using Cys and Cys-Gly selected by LASSO regression achieved an AUC of 0.860 in the discovery set. In the independent validation set, the AUC increased to 0.880 (95% CI: 0.792–0.968), with 80.0% sensitivity and 90.0% specificity (Figure 6B). The calibration curve showed close agreement between predicted probabilities and observed outcomes. The combined model performed significantly better than any single metabolite (*p* < 0.001). Overall, Cys and Cys-Gly formed the most discriminative biomarker pair for sensitive HCC diagnosis to some extent.

## 4. Discussion

BQB-derived products contain a quinolinium quaternary ammonium moiety bearing a permanent positive charge, ensuring near-quantitative ionization efficiency (effectively 100%) in solution [8,10]. Nevertheless, comparative analysis against certified reference standards demonstrates that IAM exhibits superior detection sensitivity relative to BQB. In serum sample analysis, prior studies have revealed that, in the absence of dispersive solid-phase extraction (dSPE) for sample cleanup, BQB is highly susceptible to substantial matrix interference, leading to biased quantitative results [8]. In contrast, endogenous metabolites containing alkyne functionalities are exceedingly rare, and the alkyne group imparts pronounced steric hindrance. Consequently, selective monitoring of alkyne-tagged analytes via MRM effectively excludes interference from high-abundance matrix components and side-reaction byproducts, thereby markedly enhancing method selectivity and significantly reducing reliance on dSPE steps.

Although the dSPE step was omitted, pre-analytical procedures including thiol reduction and subsequent protein precipitation as well as the overall analytical workflow may still introduce artifactual interferences such as oxidation or degradation, thereby compromising the accuracy of quantitative results. To address the inherent instability of thiol-containing compounds, particularly their propensity to undergo oxidation and form disulfide bonds, this study adopted a previously validated, optimized sample preparation protocol [8,10,15]. Specifically, TCEP was employed for the selective reduction of disulfide bonds, ensuring that free thiols remain stable in the reduced state. Concurrently, EDTA was added to the chelate free metal ions in the system, thereby effectively suppressing metal ion-mediated oxidative side reactions. Notably, thiols are more readily deprotonated under alkaline conditions, which accelerates both disulfide bond formation and oxidative degradation. To mitigate this, the TCEP-mediated reduction was rigorously conducted under acidic pH conditions and at a precisely controlled temperature of 45 °C. Protein precipitation was then performed directly following reduction, with the entire sample preparation process carried out under low-pH and tightly regulated thermal conditions. This strategy substantially minimizes the risks of oxidation and degradation, thereby markedly attenuating oxidative interference to quantitative outcomes during the pretreatment window.

Precision evaluation demonstrated that although thiol-containing analytes were present at relatively high concentrations (μM–mM range) and thus theoretically susceptible to oxidation or degradation such effects exerted negligible impact on the quantitative results obtained from real biological samples. Furthermore, to accommodate the analytical demands of complex biological matrices such as serum, the derivatization reaction was conducted within a buffered system, ensuring high reaction specificity while concurrently mitigating potential competitive side reactions and interference from hydrophobic matrix components. Although current sample preparation requires a comparatively longer duration to achieve complete and efficient thiol labeling, the elimination of dSPE-based cleanup enables practical translation into clinical settings. Specifically, high-throughput clinical applications can be realized through the development of a 96-well-plate-based direct derivatization workflow or by engineering novel, highly efficient derivatization systems, thereby significantly enhancing overall analytical throughput and robustly meeting the requirements of clinical high-throughput testing.

In addition, in actual sample analyses, age and sex were preliminarily matched. However, key clinical variables associated with non-malignant liver diseases, such as hepatic functional status, degree of liver cirrhosis, and concomitant medication use, have not yet been systematically incorporated into the control group design. Notably, various pathological processes including hepatic dysfunction and oxidative stress can induce analogous alterations in thiol metabolism. Consequently, the observed changes in thiol metabolites in this study are not solely attributable to HCC, but more likely reflect systemic perturbations in redox homeostasis and sulfur metabolism. These changes may be jointly influenced by multiple physiological and pathological factors, including hepatic impairment and other underlying liver disease states, thereby partially limiting their interpretability as HCC-specific biomarkers. Although these metabolic alterations lack disease specificity, they nevertheless exhibit statistically significant differences between HCC patients and healthy controls. They demonstrate robust discriminatory performance within statistical models for HCC patients and healthy controls, suggesting potential utility in disease status assessment and auxiliary diagnosis. Furthermore, given that the current analytical workflow entails relatively long overall processing times and relies on a limited validation cohort, future studies should prioritize large-scale, multicenter clinical validation. Such efforts should integrate comprehensive multidimensional clinical data and explicitly include non-neoplastic liver disease controls to rigorously evaluate the specificity and translational relevance of the present findings.

## 5. Conclusions

This study developed an IAM derivatization-based LC-MS method for quantification of seven thiol metabolites in human serum samples. IAM improved reversed-phase chromatographic retention of thiol metabolites and generated analyte-specific fragment ions in MS/MS analysis, enhancing their specificity in MRM detection. The method achieves pM sensitivity, high specificity, excellent reproducibility, and strong tolerance to the serum matrix. Clinical application revealed significantly lower serum levels of Cys, Hcy, GSH, and Cys-Gly in HCC patients, which is consistent across both the discovery and independent validation sets. The Cys/Cys-Gly-based two-metabolite model achieved good sensitivity and promising specificity for preliminary HCC diagnosis to some extent. In summary, this study offers a new attempt for hepatocellular carcinoma diagnosis using a thiol biomarker-based LC-MS method.

## Figures and Tables

**Table 1:** Linearities and limits of detection for the investigated thiol metabolites.

Analytes	Linear Range	Regression Data			LODs
	(nmol/L)	Slope	Intercept	R^2^ Value	(nmol/L)
Cys	0.1–2000	0.0032	0.0289	0.9998	0.03
Hcy	0.1–2000	0.0074	0.0084	0.9998	0.05
GSH	0.1–1000	0.0022	0.0078	0.9998	0.03
NAC	0.1–2000	0.0007	0.0006	0.9981	0.3
Cys-Gly	0.1–5000	0.0034	0.0158	0.9995	0.1
Glu-Cys	0.1–2000	0.0109	0.0077	0.9991	0.02
CA	0.1–100	0.1118	−0.0086	0.9995	0.02

**Table 2:** Recoveries, intra- and inter-day precisions (RSDs) for the detected thiol metabolites in human serum samples.

Thiols	Intra-Day Precision			Inter-Day Precision			Recovery (%)		
	(RSD.%; *n* = 5)			(RSD.%; *n* = 3)					
	Low	Medium	High	Low	Medium	High	Low	Medium	High
	(nmol/L)	(nmol/L)	(nmol/L)	(nmol/L)	(nmol/L)	(nmol/L)	(nmol/L)	(nmol/L)	(nmol/L)
Cys	0.7	8.2	7.5	3.3	1.9	2.7	93.7 ± 8.4	86.4 ± 8.9	102.8 ± 7.4
Hcy	3.0	6.6	1.5	3.2	2.5	5.3	96.6 ± 5.1	88.6 ± 8.2	85.9 ± 5.3
GSH	2.4	6.8	4.4	4.5	10.2	5.3	102.9 ± 9.3	88.4 ± 7.8	80.4 ±6.6
NAC	7.9	4.2	5.6	7.8	6.6	3.2	88.4 ± 8.7	81.1 ± 7.2	99.2 ± 10.3
Cys-Gly	8.1	6.6	1.8	1.5	10.7	3.6	82.7 ± 3.7	81.2 ± 9.2	105.1 ± 4.2
Glu-Cys	2.1	3.3	2.3	2.0	3.8	3.9	85.1 ± 8.4	98.6 ± 4.4	96.4 ± 4.5
CA	5.3	8.9	2.3	1.5	5.0	2.1	86.8 ± 6.9	95.4 ± 9.5	99.4 ± 6.2

**Table 3:** Summary of the methods for determination of thiols in the trans-sulfuration pathway from human serum samples.

Analytical Methods	Derivatization	LODs (nmol/L)							Reference
		Cys	Hcy	NAC	GSH	CysGly	GluCys	CA	
LC-MS	NCS-OTPP ^a^	19.2	28.8	-	57.6	-	-	-	[17]
LC-MS	BQB	6.0	1.6	0.7	1.9	3.2	2.1	4.0	[8]
LC-MS	-	-	74.00	-	-	-	-	-	[13]
LC-MS	NEM ^ab^	1000	100	-	3000	300	300	-	[19]
LC-MS	mBrB ^a^	-	-	0.31 fmol	-	4.98 fmol	-	-	[26]
LC-MS	Br-OTPP ^a^	7.5 fmol	9 fmol	0.8 fmol	8 fmol	7.5 fmol	8 fmol	8 fmol	[12]
MALDI-MS	Br-C60 ^a^	180	140	-	70	120	-	-	[15]
LC-MS	IAM	0.03	0.05	0.03	0.30	0.10	0.02	0.02	This work

^a^ MALDI-MS—matrix-assisted laser desorption/ionization time-of-flight mass spectrometry; NCS-OTPP—(R)-(5-(3-isothiocyanatopyrrolidin-1-yl)-5-oxopentyl) triphenylphosphonium; NEM—N-ethylmaleimide; mBrB—monobromobimane; Br-C60—bromoacetyl-functionalized C60. ^b^ data from lowest limit of quantification.

## Data Availability

The original contributions presented in this study are included in the article and Supplementary Materials. Further inquiries can be directed to the corresponding authors.

## Supplementary Materials

The following supporting information can be downloaded at https://www.mdpi.com/article/10.3390/metabo16050345/s1, Figure S1: Reaction optimization; Figure S2: Matrix effect; Table S1: MRM transitions of IAM derivatives; Table S2: LODs of investigated metabolites; Table S3: Concentration of thiol metabolites in human serum samples; Table S4: Results of difference test for discovery set; Table S5: Results of difference test for validation set.
