# Supplementary material for: Iodoacetamine-Alkyne Derivatization-Based Liquid Chromatography–Mass Spectrometry Method for Quantification of Thiol Metabolites in Serum Samples of Hepatocellular Carcinoma Patients
Source: Metabolites. 2026 May 20;16(5):345. doi: 10.3390/metabo16050345 (PMC13209086; doi:10.3390/metabo16050345)

SUPPORTING INFORMATION

Iodoacetamine-alkyne derivatization based liquid chromatography mass spectrometry method for quantification of thiol metabolites in serum samples of hepatocellular carcinoma patients

Chun Mei [1], Xin-Ze Wu [1], Hua-Ming Xiao [2*], Azamat Temerdashev [3], Na An [2], Quan-Fei Zhu[2] and Yu-Qi Feng [1,2,*]

[1] Department of Chemistry, Wuhan University, Wuhan 430072, China; chunmei@whu.edu.cn (C.M.); wuxinze@whu.edu.cn (X.-Z.W.)

[2] School of Biomedical Engineering and Health, Wuhan Textile University, Wuhan 430200, China; hmxiao@wtu.edu.cn (H.-M.X.); naan_anna@whu.edu.cn (N.A.); qf_zhu@whu.edu.cn (Q.-F.Z.); yqfeng@whu.edu.cn (Y.-Q.F.)

[3] Analytical Chemistry Department, Kuban State University, Krasnodar 350040, Russia; TemerdashevAZ@gmail.com (A. T.)

* Correspondence: hmxiao@wtu.edu.cn (H.-M.X.); yqfeng@whu.edu.cn (Y.-Q.F.)

LIST OF CONTENTS

SUPPORTING FIGURES AND TABLES

Table S1. MRM transitions of IAM derivatives of thiol metabolites. S3

Table S2. LODs of RSH, IAM derivatives, and BQB derivatives...................................S4

Table S3. Concentration of thiol metabolites in human serum samples from hepatocellular carcinoma patients and healthy controls. S5

Table S4. Median concentrations, interquartile ranges, and results of difference tests for seven thiol compounds between the HCC and CTL groups (discovery set). S7

Table S5. Median concentrations, interquartile ranges, and results of difference tests for seven thiol metabolites between the HCC and CTL groups (validation set).........S8

Figure S1. Optimization of IAM derivatization reaction conditions, including (A) pH values of reaction towards thiols, (B) pH values of reaction towards amine-containing compounds, (C) reaction temperature, and (D) reaction time. S9

Figure S2. Matrix effect of thiol metabolites in human serum samples. S10

SUPPORTING FIGURES AND TABLES

Table S1. MRM transitions of IAM derivatives of thiol metabolites.

Analytes	Scan mode	Retention time (min)	Precursor ion (Da)	Product ion (Da)	CE (V)
Cys	+	1.6	259.1	170.2, 213.2	-17, -12
Hcy	+	2.2	273.1	56.2, 172.2	-21, -17
GSH	+	2.7	445.2	316.2, 213.2	-16, -21
NAC	+	3.5	301.1	259.1, 196.2	-14, -15
Cys-Gly	+	1.9	31 6.1	299.1, 170.1	-14, -20
Glu-Cys	+	2.6	388.1	249.1, 291.1	-26, -28
CA	+	1.5	215.1	198.1, 170.1	-13, -16
d4-Hcy	+	2.2	277.1	60.1, 231.1	-18, -15

Table S2. LODs of RSH, IAM derivatives, and BQB derivatives.

Analytes	LODs (nmol/L)				
	Unlabeled	IAM labeled	Improved Folds	BQB labeled	Improved Folds
Cys	5	0.03	160	0.3	16
Hcy	2	0.05	40	0.1	20
GSH	1	0.03	33	0.1	10
NAC	10	0.3	33	0.2	50
Cys-Gly	8	0.1	80	0.3	26
Glu-Cys	2	0.02	100	0.3	6
CA	1	0.02	50	0.2	5

Table S3. Concentration of thiol metabolites in human serum samples from hepatocellular carcinoma patients and healthy controls.

Samples	Cys (μmol/L)		Hcy (μmol/L)		GSH (μmol/L)		NAC (nmol/L)		Cys-Gly (μmol/L)		Glu-Cys (μmol/L)		CA (nmol/L)	
	CTL	HCC	CTL	HCC	CTL	HCC	CTL	HCC	CTL	HCC	CTL	HCC	CTL	HCC
Test 1	192.4	164.7	9.4	4.9	1.2	1.1	97.8	55.0	35.6	37.6	1.5	1.8	8.5	9.1
Test 2	197.0	168.7	13.3	5.1	1.1	0.2	57.7	70.5	27.2	13.7	1.5	1.6	7.6	9.8
Test 3	189.0	157.8	7.5	3.9	1.6	0.2	320.0	104.2	28.2	12.3	1.7	1.3	17.8	7.6
Test 4	221.4	224.9	13.3	5.7	6.2	0.3	101.4	82.4	59.2	19.1	2.1	1.8	18.9	6.7
Test 5	214.0	224.1	7.3	11.6	1.4	0.3	99.6	104.8	49.0	15.0	1.8	1.7	7.4	11.4
Test 6	174.4	133.2	5.1	5.3	0.4	0.7	74.2	59.4	17.6	30.6	1.1	1.3	9.0	18.4
Test 7	199.2	169.1	10.1	7.0	1.2	0.2	62.4	91.0	36.5	21.5	1.3	1.7	19.8	9.1
Test 8	187.6	150.4	6.8	4.7	0.6	0.9	91.2	104.2	41.8	34.0	1.8	1.7	9.2	8.6
Test 9	183.1	144.5	7.6	6.0	3.1	0.8	299.5	85.0	26.5	11.7	1.8	1.7	8.3	10.1
Test 10	166.2	128.8	4.7	5.6	1.8	0.6	90.3	60.5	36.7	26.8	1.6	1.4	64.5	6.3
1	186.3	168.5	10.3	8.1	0.4	3.9	57.6	136.8	13.1	24.8	1.3	0.9	9.2	9.0
2	180.2	190.3	3.3	5.9	0.8	1.2	103.6	74.2	25.1	39.5	1.6	1.7	23.4	9.6
3	245.8	137.9	12.0	5.5	1.3	2.9	74.5	64.9	20.0	29.8	1.6	2.0	8.7	9.9
4	249.5	129.4	10.7	4.6	2.1	0.6	107.4	65.3	32.3	14.7	1.4	1.2	13.7	15.9
5	196.9	165.3	21.3	8.9	0.8	0.4	85.1	87.4	29.0	15.1	1.3	1.4	15.9	9.1
6	185.2	169.7	8.3	11.1	1.4	1.9	86.8	14.3	35.6	29.8	1.5	1.2	12.5	7.9
7	171.9	194.0	3.2	6.4	0.4	0.3	52.8	88.6	22.5	9.4	1.3	1.7	12.1	53.1
8	168.1	200.7	8.9	6.9	0.9	0.9	57.6	88.5	31.1	20.9	1.3	1.8	11.8	9.0
9	190.2	167.2	8.8	9.2	0.3	1.0	65.0	62.7	21.5	35.1	1.4	1.7	14.0	10.2
10	164.9	208.3	4.2	10.7	0.7	0.3	70.9	90.4	22.7	5.9	1.3	1.7	18.4	9.8

(To be continued)

Samples	Cys (μmol/L)		Hcy (μmol/L)		GSH (μmol/L)		NAC (nmol/L)		Cys-Gly (μmol/L)		Glu-Cys (μmol/L)		CA (nmol/L)	
	CTL	HCC	CTL	HCC	CTL	HCC	CTL	HCC	CTL	HCC	CTL	HCC	CTL	HCC
11	210.0	151.3	6.4	5.9	0.8	0.9	87.4	330.2	33.2	31.9	1.4	1.6	10.4	8.1
12	217.4	148.9	9.1	4.1	0.6	0.4	298.6	44.8	30.5	19.3	1.3	1.0	8.3	8.0
13	221.4	148.8	12.1	2.9	1.8	0.5	81.9	269.9	32.9	8.8	1.4	1.6	11.5	9.5
14	197.5	164.6	11.1	8.1	7.1	0.4	90.8	67.0	61.9	19.8	2.4	1.1	11.8	9.7
15	214.6	149.6	7.0	3.7	0.7	0.4	76.6	47.9	19.1	27.7	1.4	1.3	14.1	9.7
16	184.4	176.9	5.3	10.8	0.2	0.6	82.3	97.5	16.6	12.9	1.5	1.9	20.1	7.3
17	204.6	152.3	9.2	10.9	1.1	2.8	307.7	476.5	18.5	26.3	1.6	1.0	6.8	8.2
18	173.2	192.5	5.6	11.7	0.7	6.9	77.8	126.2	22.6	38.2	1.1	1.0	9.3	19.7
19	233.5	144.0	15.0	5.8	2.1	0.9	92.7	345.0	37.9	19.9	1.4	1.6	13.0	8.6
20	184.3	182.7	7.9	6.2	2.3	0.2	309.0	44.6	36.2	9.7	1.5	1.3	8.5	8.4
21	236.2	143.9	9.0	8.9	0.6	0.3	956.4	168.5	28.6	15.8	1.0	1.9	24.8	8.6
22	168.0	203.4	8.0	4.0	0.7	0.7	59.5	73.1	22.3	21.8	1.2	1.1	7.1	10.7
23	212.9	149.7	11.0	3.7	1.4	0.2	98.5	63.8	27.1	11.1	1.6	1.5	8.9	9.0
24	198.2	154.0	10.8	6.8	1.4	0.6	523.7	68.9	22.4	21.6	1.3	1.4	9.7	31.8
25	197.5	161.5	12.8	7.0	0.9	1.2	94.8	79.1	19.8	21.9	1.1	1.6	12.8	9.6
26	176.9	192.2	7.4	6.5	5.2	0.8	129.9	92.8	29.5	10.8	1.4	1.3	9.1	8.8
27	247.9	133.7	58.0	7.0	1.0	0.7	132.6	19.7	30.8	22.0	1.9	1.3	10.6	7.1
28	180.4	190.2	11.0	5.4	1.3	0.7	76.4	94.9	20.0	28.6	1.5	1.6	7.8	6.5
29	256.4	117.6	16.9	3.9	1.2	0.4	109.0	91.2	39.3	10.3	1.6	1.7	7.3	9.7
30	172.1	193.0	7.3	7.3	1.4	0.5	44.2	78.9	35.5	13.1	1.3	1.8	14.3	10.2

Table S4. Median concentrations, interquartile ranges, and results of difference tests for seven thiol compounds between the HCC and CTL groups (discovery set).

Analytes	Shapiro-Wilk test	Median (P_{25}, P_{75})		Mann-Whitney U test
		CTL	HCC	
Cys	0.000**	197.205 (179.397, 218.382)	164.941 (148.887, 190.792)	0.000**
Hcy	0.000**	9.039 (7.214, 11.321)	6.665 (5.189, 8.872)	0.004**
GSH	0.000**	0.986 (0.677, 1.402)	0.658 (0.378, 1.057)	0.032*
NAC	0.48	0.087 (0.074, 0.114)	0.083 (0.065, 0.105)	0.375
Cys-Gly	0.018*	27.857 (21.135, 32.935)	20.378 (12.421, 27.953)	0.003**
Glu-Cys	0.13	1.390 (1.302, 1.534)	1.514 (1.202, 1.735)	0.433
CA	0.000**	0.012 (0.009, 0.014)	0.009 (0.008, 0.010)	0.055

* $p<0.05$ ** $p<0.01$

Table S5. Median concentrations, interquartile ranges, and results of difference tests for seven thiol metabolites between the HCC and CTL groups (validation set).

Analytes	Shapiro-Wilk test	Median (P_{25}, P_{75})		Mann-Whitney U test
		CTL	HCC	
Cys	0.636	190.674 (180.905, 202.887)	161.223 (141.690, 182.823)	0.034*
Hcy	0.008**	7.576 (6.389, 10.877)	5.441 (4.874, 6.244)	0.028*
GSH	0.000**	1.290 (0.964, 2.088)	0.482 (0.231, 0.830)	0.003**
NAC	0.000**	0.095 (0.071, 0.151)	0.084 (0.060, 0.104)	0.364
Cys-Gly	0.427	36.049 (27.005, 43.616)	20.284 (13.383, 31.466)	0.019*
Glu-Cys	0.646	1.679 (1.450, 1.808)	1.670 (1.423, 1.737)	0.65
CA	0.000**	0.009 (0.008, 0.019)	0.009 (0.007, 0.010)	0.45

* $p<0.05$ ** $p<0.01$

Figure S1. Optimization of IAM derivatization reaction conditions, including (A) pH values of reaction towards thiols, (B) pH values of reaction towards amine-containing compounds, (C) reaction temperature, and (D) reaction time.

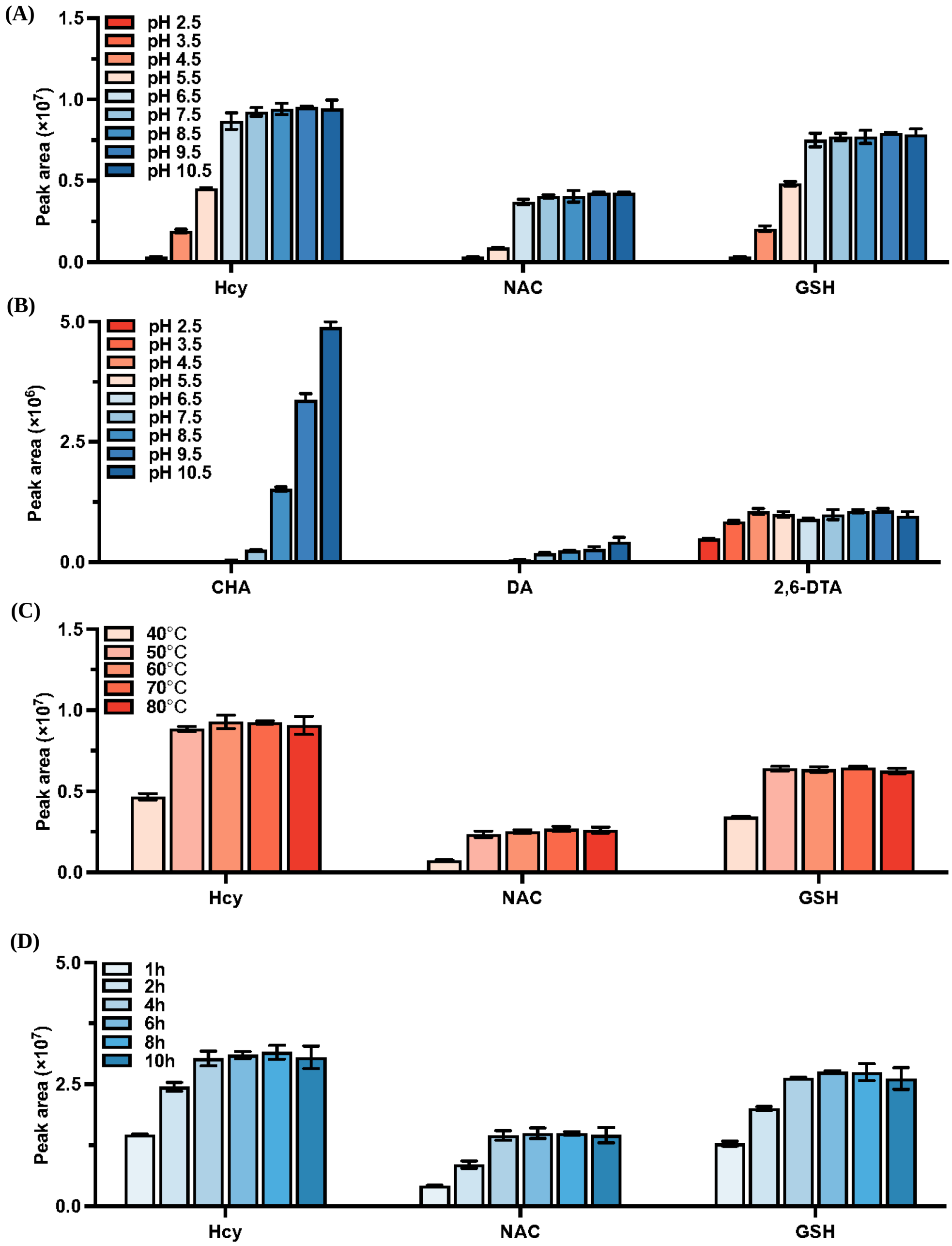

Figure S2. Matrix effect of thiol metabolites in human serum samples.

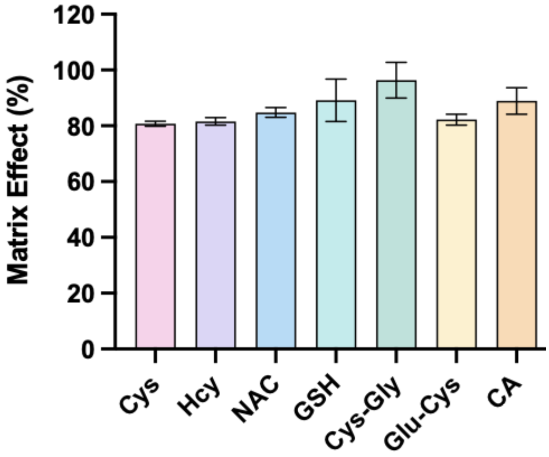

Supplement: Supplementary file 1 [file metabolites-16-00345-s001.zip › Supporting Information.pdf]
